# *Cypripedium subtropicum* embryo development and cytokinin requirements for asymbiotic germination

**DOI:** 10.1186/s40529-022-00359-4

**Published:** 2022-09-30

**Authors:** Holger Perner, Rong Zhou, Wenqing Perner, Hong Jiang, Yung-I. Lee

**Affiliations:** 1 Hengduan Mountains Biotechnology Ltd, Chengdu, 6100225 Sichuan China; 2 Yunnan Laboratory for Conservation of Rare, Public Key Laboratory of the National Forestry and Grassland Administration, Yunnan Academy of Forestry and Grassland, Endangered & Endemic Forest Plants, Kunming, 650201 Yunnan China; 3 Department of Life Science, National Taiwan University, Taipei, 10617 Taiwan; 4 Institute of Ecology and Evolutionary Biology, National Taiwan University, Taipei, 10617 Taiwan

**Keywords:** Asymbiotic germination, Embryogenesis, Cytokinins, Seed coat, Lady’s slipper orchids

## Abstract

**Background:**

*Cypripedium subtropicum* is a unique, endangered lady’s slipper orchid with evergreen leaves on non-dormant shoots that is native to southwestern China. This study documents the major developmental events in *C. subtropicum* seed development from fertilization to seed maturity, determines the optimum period for seed collection, and examines the cytokinin requirements for asymbiotic germination and protocorm survival.

**Results:**

Structural studies revealed that embryo development proceeded after successful fertilization at 60 days after pollination (DAP). At 105 DAP, a globular embryo with the shrinking inner seed coat was observed, and seeds collected at this time point exhibited optimal germination. After 120 DAP, most seeds had a mature embryo within the capsule, and within the cells of the embryo proper, numerous proteins/lipid bodies were present as the main storage products. In addition, the inner seed coat had compressed into a thin layer that tightly enclosed the embryo, while the outer seed coat had progressively elongated, resulting in a hair-like appearance of the mature seed. Histochemical staining using Nile red and toluidine blue O (TBO) indicated that the lignified inner and outer seed coats may lead to coat-imposed dormancy. Seeds collected at this stage germinated poorly. Analyses of cytokinin preferences and optimal concentrations for germination and protocorm survival showed that both 6-(γ,γ-dimethylallylamino) purine (2iP) and 6-benzylaminopurine (BA) enhanced germination compared with the control, although higher concentrations of BA (4 and 8 μM) suppressed germination. The protocorm survival rate improved with increasing 2iP concentration.

**Conclusions:**

This study provides a reproducible procedure for culturing immature seeds of *C. subtropicum* based on a defined time schedule of seed development. In addition, the cytokinin 2iP was shown to improve germination and protocorm survival. This study provides a scientific basis for seedling establishment through asymbiotic seed culture for further reintroduction efforts.

## Background

The genus *Cypripedium* comprises approximately 50 species that are grouped into ten sections based on molecular phylogenetic analyses (Li et al. 2011). This genus has a broad geographical distribution from subtropical to temperate latitudes in the Northern Hemisphere (Cribb 1997). *Cypripedium* species have attractive flowers with a distinctive labellum resembling a slipper or shoe, giving rise to the common name of lady’s slipper orchids. Several *Cypripedium* species are endangered due to shrinking natural habitats and illegal collection for horticultural markets. Among *Cypripedium* species, *Cypripedium subtropicum* (section *Subtropica*) is probably one of the most poorly characterized species. It was initially described from an herbarium specimen collected in Medog County in southeastern Tibet (Chen and Lang 1986). In 2009, similar live plants were found in subtropical/tropical forests in southeastern Yunnan Province and adjacent Vietnam, more than 1000 km from the collection site of the original type specimen (Jiang and Liu 2009; Averyanov et al. 2017). The flowers of *C. subtropicum* visually mimic an aphid-colonized labellum and chemically mimic aphid alarm pheromones to attract hoverflies for pollination (Jiang et al. 2020). Despite its relatively broad distribution, *C. subtropicum* is among the endangered (EN) species on the Global Red List of the International Union for Conservation of Nature (IUCN) (Rankou and Averyanov 2014). Our field explorations and a report from Averyanov et al. (2017) confirm that this species is indeed at risk of extinction because of overcollection of several known populations.

To conserve this endangered species and enable commercial production, a practical and efficient in vitro propagation system is desirable. In contrast to most epiphytic orchids, in vitro seed germination of terrestrial orchids remains challenging (Arditti et al. 1982; Rasmussen 1995). Culturing immature seeds before dormancy is one approach to improve the seed germination of terrestrial orchids (Linden 1980; Light and MacConaill 1998; Yamazaki and Myoshi 2006). Successful culture of immature seeds has been documented for some *Cypripedium* species (St-Arnaud et al. 1992; De Pauw and Remphrey 1993), but the timing of seed collection and culture requirements may be species specific (Zeng et al. 2014). Our previous studies of *Cypripedium* species have shown that basic knowledge of embryo and seed development is critical for designing seed germination experiments (Lee et al. 2005; Hsu and Lee 2012; Zhang et al. 2013; Jiang et al. 2017). Consequently, the objectives of the present study were to determine the optimum period for the collection of *C. subtropicum* seeds to maximize germination in vitro and the optimum concentration and composition of cytokinins in the culture medium for asymbiotic germination and protocorm survival.

## Methods

### Plant materials

**Fig. 1:**
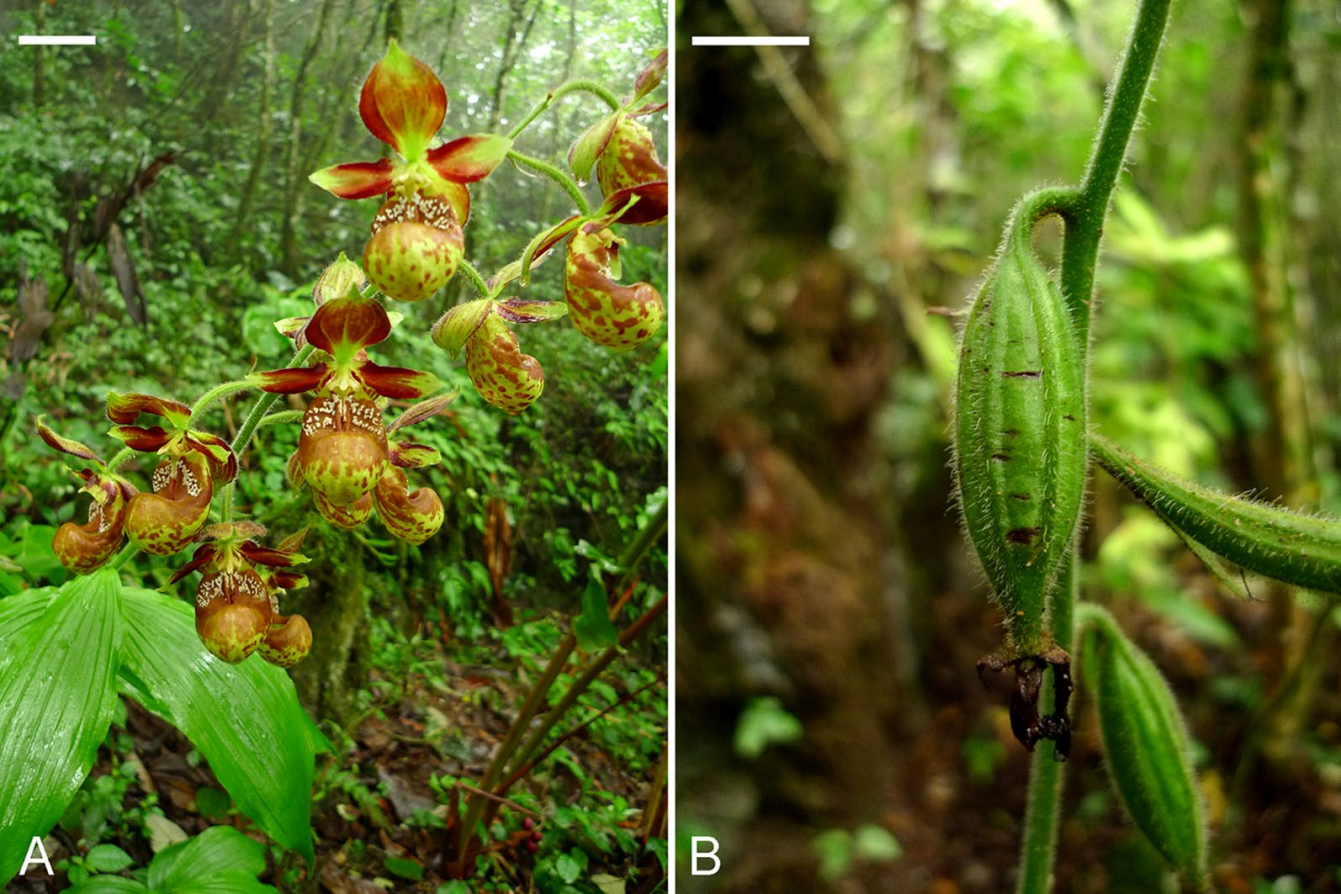
**A** Flowers of *C. subtropicum*. Scale bar = 2 cm. **B** Capsules at 120 DAP. Scale bar = 1 cm

Plants of *C. subtropicum* were maintained in their natural habitat in Malipo County, Yunnan Province, China. To guarantee a good capsule set and seed quantity, flowers were self-pollinated manually at the time of full bloom in early July (Fig. 1A). Developing capsules (Fig. 1B) were harvested at 15 day intervals after pollination for subsequent experiments.

### Light microscopy

The developing capsules were sliced and fixed immediately for 12 h at room temperature in 1% glutaraldehyde in 0.1 M phosphate buffer, pH 6.8. The fixed samples were then dehydrated in an ethanol series and embedded in Technovit 7100 (Kulzer & Co., Germany) as described by Yeung and Chan (2015). Serial, 3 µm-thick sections were cut using a Reichert-Jung 2040 Autocut rotary microtome with glass knives. The historesin sections were collected on slides for periodic acid–Schiff (PAS) staining, followed by counterstaining with either 0.1% (*w/v*) toluidine blue O (TBO) in benzoate buffer for general histology or 1% (*w/v*) amido black 10B in 7% acetic acid solution (Yeung 1984). Cuticular material was stained using Nile red as described by Lee et al. (2006). In brief, the sections were stained with 1 μg ml^−1^ Nile red (Sigma Chemical Co., St. Louis, MO) for 1 min, washed in distilled water for 2 min, and mounted in Vectashield^®^ anti-fading medium (Vector Laboratories, Inc., Burlingame, CA). The fluorescent signal was examined using an epifluorescence microscope (Axioskop 2, Carl Zeiss AG) equipped with a Zeiss filter set. Images were captured digitally using a CCD camera attached to the microscope.

### Effect of seed collection timing

To evaluate the effect of seed collection timing, three capsules were collected every 15 days between 60 and 150 days after pollination (DAP). The capsules were surface sterilized for 15 min with 1% sodium hypochlorite solution plus a drop of wetting agent (Tween 20). After surface sterilization, the capsules were cut open, and the seeds were removed with tweezers in a laminar flow hood. Then, the seeds were inoculated onto the surface of 10 mL of solidified medium dispensed into a 20 × 100 mm culture tube. The basic medium was the modified Norstog medium (Norstog 1973) described by De Pauw and Remphrey (1993), supplemented with 1 mg l^–1^ malic acid and 20 g l^−1^ sucrose and solidified with 7 g l^−1^ agar (Sigma-Aldrich Co.). The pH of the medium was adjusted to 5.7 before autoclaving at 121 °C for 15 min.

### Effect of cytokinins on asymbiotic germination

Seeds collected at 105 DAP were used to investigate the influence of cytokinins on asymbiotic germination. In this experiment, modified Norstog medium was supplemented with either 6-(γ,γ-dimethylallylamino)purine (2iP, Sigma-Aldrich Co.), 6-benzylaminopurine (BA, Sigma-Aldrich Co.) or kinetin (KN, Sigma-Aldrich Co.) at concentrations of 1, 2, 4 and 8 μM. Modified Norstog medium without cytokinin supplementation was used as a control.

### Culture conditions and evaluation of germination and survival percentage

After sowing, the culture tubes were incubated in a growth chamber at 25 ± 1 °C and constant darkness. Each culture tube was examined at 15 day intervals for 6 months by using a stereomicroscope. Emergence of the embryo from the seed coat was considered germination. The germination percentage was calculated by dividing the number of germinated seeds by the total number of seeds and multiplying by 100. The survival percentage was calculated by dividing the number of live protocorms by the number of germinated protocorms and multiplying by 100.

### Greenhouse acclimatization and seedling growth

The seedlings (about 2 cm in height) were taken out from flasks, then transplanted to clay pots filled with the potting mix consisting of 1 part of pumice and 2 parts of bark. The seedlings were grown in the greenhouse shaded by nylon sunshade net to give 75% of full sunlight, and the air temperature in the greenhouse ranged from 10 to 25 °C

### Experimental design and data analysis

In each experiment, three capsules were collected for inoculation. The seeds from each capsule were evenly sown into the replicates of each treatment. Each treatment comprised twelve culture tubes (replicates) and was conducted three times. Experiments were performed in a completely randomized design. The data were statistically analyzed using analysis of variance (ANOVA). The means were separated using Fisher’s protected least significant difference test at *P* < 0.05 (SAS statistical software, version 8.2, SAS Institute, Cary, NC).

## Results

### Embryo development

**Fig. 2:**
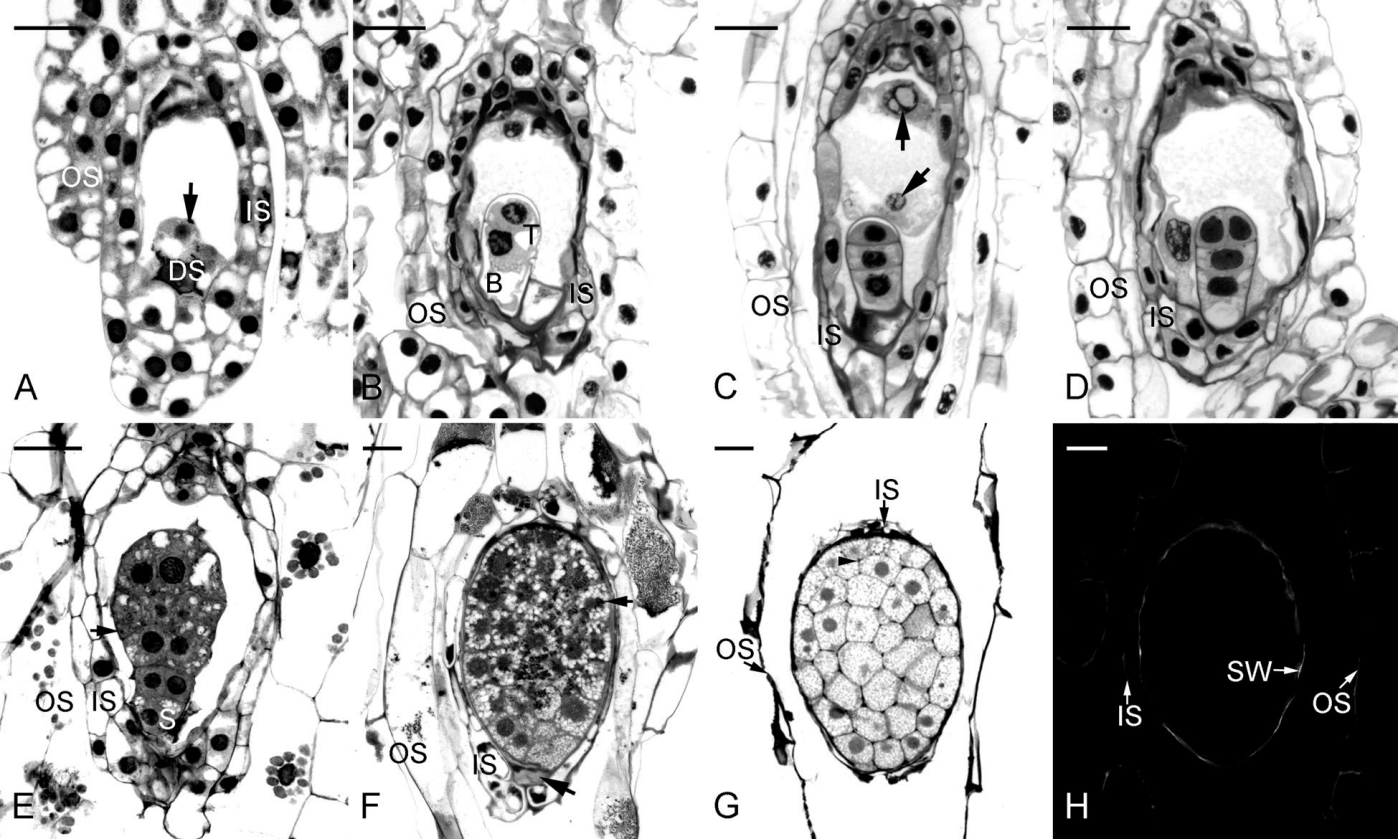
Light micrographs of developing seeds of *C. subtropicum*. **A** The zygote (arrow) after fertilization at 45 DAP. At the micropylar end, one synergid has degenerated (DS). Inner seed coat (IS), outer seed coat (OS). Scale bar = 30 µm. **B** The first cell division of the zygote results in the formation of a smaller terminal cell (T) and a larger basal cell (B). Inner seed coat (IS), outer seed coat (OS). Scale bar = 30 µm. **C** A three-cell embryo. Primary endosperm nuclei (arrows) are observed within the cavity, but the endosperm fails to develop and eventually degenerates. Inner seed coat (IS), outer seed coat (OS). Scale bar = 30 µm. **D** The terminal cell of a three-cell embryo divides vertically, resulting in the formation of a T-shaped, four-cell embryo. Inner seed coat (IS), outer seed coat (OS). Scale bar = 30 µm. **E** The embryo proper continues to develop by cell division to form an early globular embryo. The suspensor (S) of this species consists of a single cell and does not further enlarge. At this stage, the cytoplasm in cells of the embryo proper appears dense, and a few tiny starch grains can be observed within the cytoplasm, indicating the accumulation of storage products. Inner seed coat (IS), outer seed coat (OS). Scale bar = 30 µm. **F** Longitudinal section of a globular embryo. At this stage, the suspensor is degenerating (arrow), and the cells of the inner seed coat (IS) and outer seed coat (OS) are beginning to degenerate and dehydrate. Several protein bodies (arrow) are present in the cells of the embryo proper. Scale bar = 30 μm. **G** Longitudinal section of a mature seed. At maturity, the embryo is enveloped by the shriveled inner seed coat (IS) and the outer seed coat (OS). Starch grains are apparent, and a few tiny protein bodies (arrow) can be observed within the embryo proper. Although lipids cannot be preserved in historesin, the abundant translucent vesicles within the cytoplasm of the embryo proper indicate the deposition of lipid bodies. Scale bar = 30 μm. **H** Fluorescent outline of a mature seed at the same stage shown in Fig. 2G after Nile red staining. The surface wall (SW) of the embryo proper reacts positively, while the compressed inner seed coat (IS) and outer seed coat (OS) react weakly. Scale bar = 30 μm

Table 1 summarizes the major microscopic events during seed development of *C. subtropicum* from pollination to seed maturity (Fig. 1). Fertilization occurred in most ovules at 60 DAP (Fig. 2A). The first division of the zygote was unequal and produced a smaller terminal cell and larger basal cell (Fig. 2B). The terminal cell had dense cytoplasm, whereas the basal cell was elongated and vacuolated. At 75 DAP, proembryos with different numbers of embryonic cells were observed. The basal cell of the two-cell embryo then divided transversely, giving rise to a three-cell embryo (Fig. 2C). The endosperm failed to develop in this species. After fertilization, the nuclei within the primary endosperm cell did not undergo further division (Fig. 2C and D), and the content of the cell was eventually absorbed by the expanding embryo. Subsequent anticlinal division in the terminal cell produced a four-cell embryo (Fig. 2D). In the four-cell embryo, the terminal tier of cells toward the chalazal end continued to divide and resulted in the formation of the embryo proper. By 90 DAP, early globular embryos were readily observed (Fig. 2E). By 105 DAP, mitotic division had ceased within the embryo proper, and large vacuoles began to be replaced by small ones (Fig. 2F). At this stage, the storage materials began to appear in the basal part of the embryo proper. At maturity (after 135 DAP), the embryo proper was approximately eight cells long and six cells across at its widest point (Fig. 2G). Within the cells of the mature embryo proper, protein and lipid bodies were the major storage products. Nile red staining indicated the presence of cuticular substances in the surface wall of the embryo proper at maturity (Fig. 2H). At 150 DAP, the capsules split to release seeds.

**Table 1:** Major microscopic structural events occurring in the developing pods of *C. subtropicum* after hand pollination

DAP	developmental stage	Integument/seed coat color
30	Megasporogenesis	White
45	Megagametogenesis	White
60	Fertilization	White
75	Proembryo stage	White
90	Early globular embryo stage	Yellowish white
105	Late globular embryo stage	A mixture of white and light brown seeds
120	Near mature seeds with degenerating suspensors	Brown
135	Dry and mature seeds	Brown
150	Capsule ripe and split	Brown

*DAP * days after pollination

### Seed coat development

**Fig. 3:**
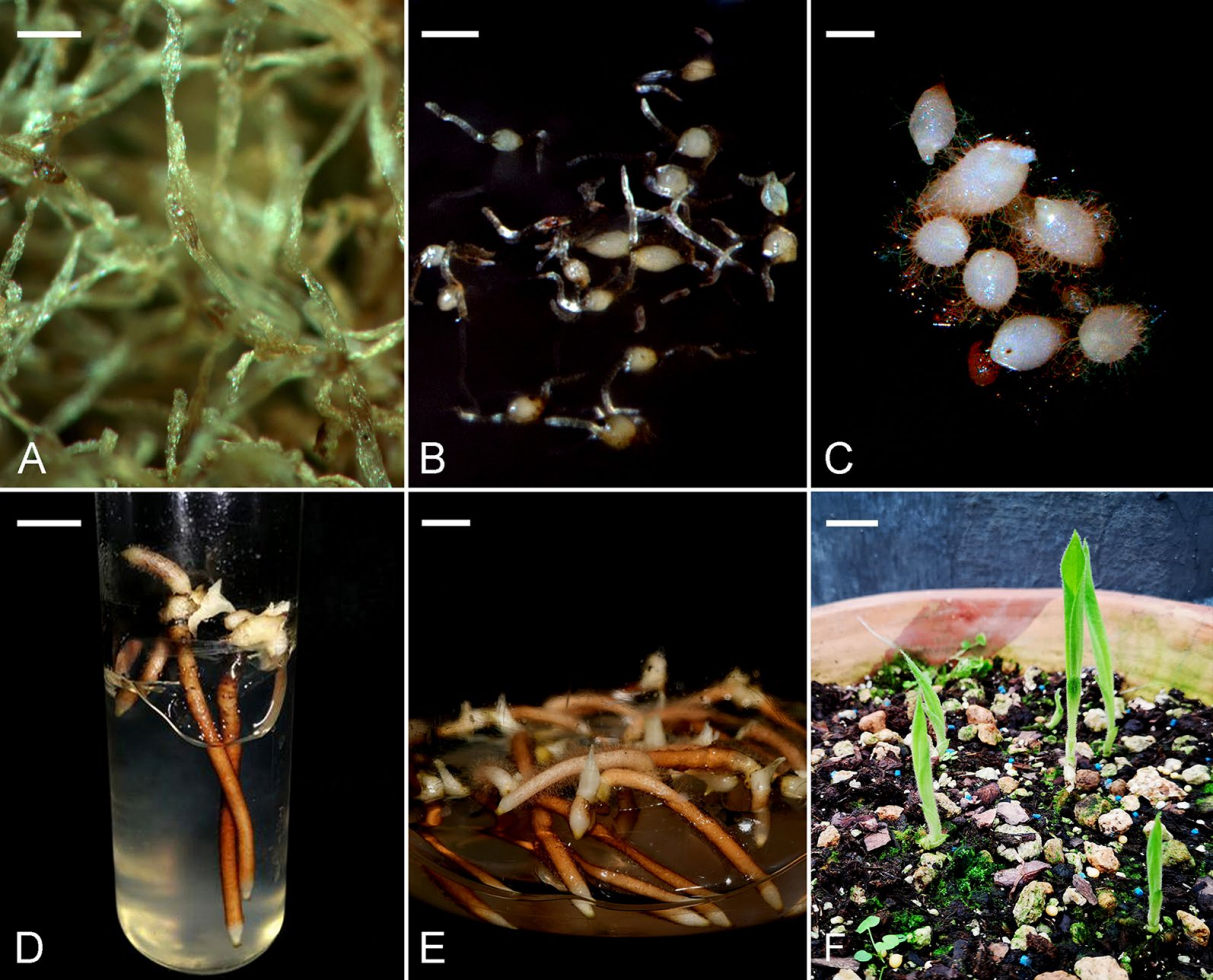
Seed germination and seedling development of *C. subtropicum*. **A** Mature seeds. Scale bar = 1 mm. **B** Swelling of the embryos results in seed coat rupture. Scale bar = 2 mm. **C** The protocorms have enlarged, and shoot tips are apparent. Scale bar = 2 mm. **D** Before shoot elongation, several roots protrude and grow quickly. Scale bar = 3 mm. **E** Shoot buds with numerous developing roots after 4 months of culture. Scale bar = 7 mm. **F** After removal from the flasks, the shoot buds are elongated with expanding leaves. Scale bar = 10 mm

At the time of fertilization, the inner seed coat enclosed the embryo sac, while the cells of the outer seed coat were still dividing and did not cover the inner seed coat (Fig. 2A). During the early stages of embryo development, the outer seed coat enclosed the embryo sac completely and continued to elongate progressively (Fig. 2B–E). In the late globular stage, the cells of the inner seed coat gradually compressed (Fig. 2F). As the seed approached maturity, both the inner and the outer seed coats became dehydrated and compressed into a thin layer (Fig. 2G). TBO staining of the radial walls of the outermost layer of the seed coat gave a greenish blue color, indicating the presence of phenolic compounds in the wall (lignification of the cell wall). In addition, the secondary walls reacted weakly with Nile red stain (Fig. 2H). The extensively elongated outer seed coat gave the seeds a hair-like appearance (Fig. 3A).

### Influence of seed collection timing on asymbiotic germination

**Fig. 4:**
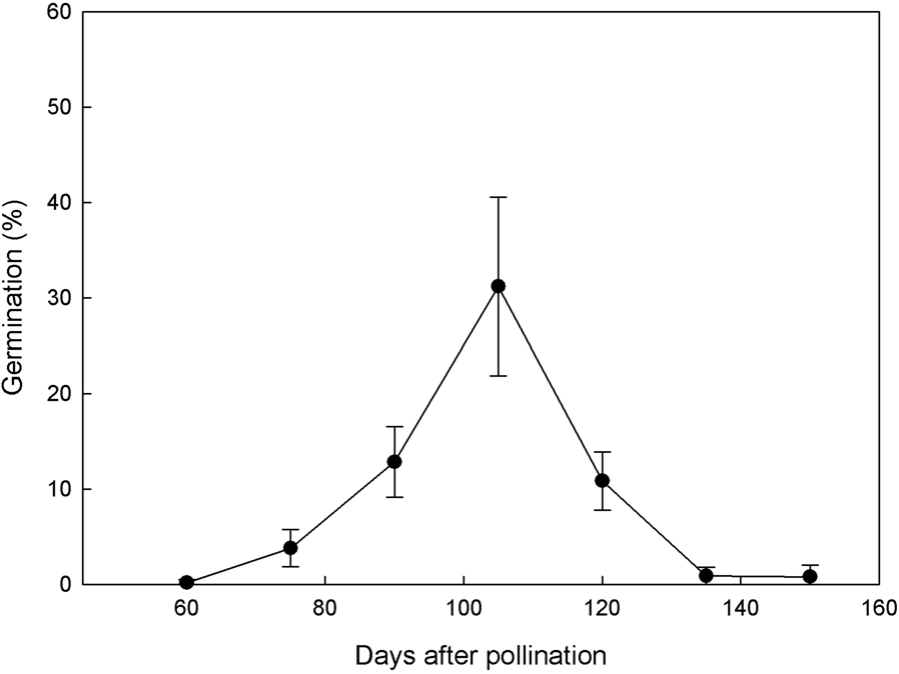
Mean percent germination of *C. subtropicum* seeds collected at successive 15 day intervals after pollination on Norstog medium. Data were scored after 6 months of culture. Error bars represent SE (n = 3)

The timing of *C. subtropicum* seed collection clearly affected the germination percentage (Fig. 4). The germination percentage was highest (31.21%) for seeds collected at 105 DAP. Very little germination (0.18–3.8%) of seeds collected at 60 or 75 DAP was observed. Germination progressively increased for seeds collected at 90 DAP (12.84%) and 105 DAP but then decreased for seeds collected at 120 DAP (10.86%). The germination percentage of seeds collected at 135 DAP or later was poor (less than 1%).

### Influence of cytokinins on asymbiotic germination and protocorm survival

**Fig. 5:**
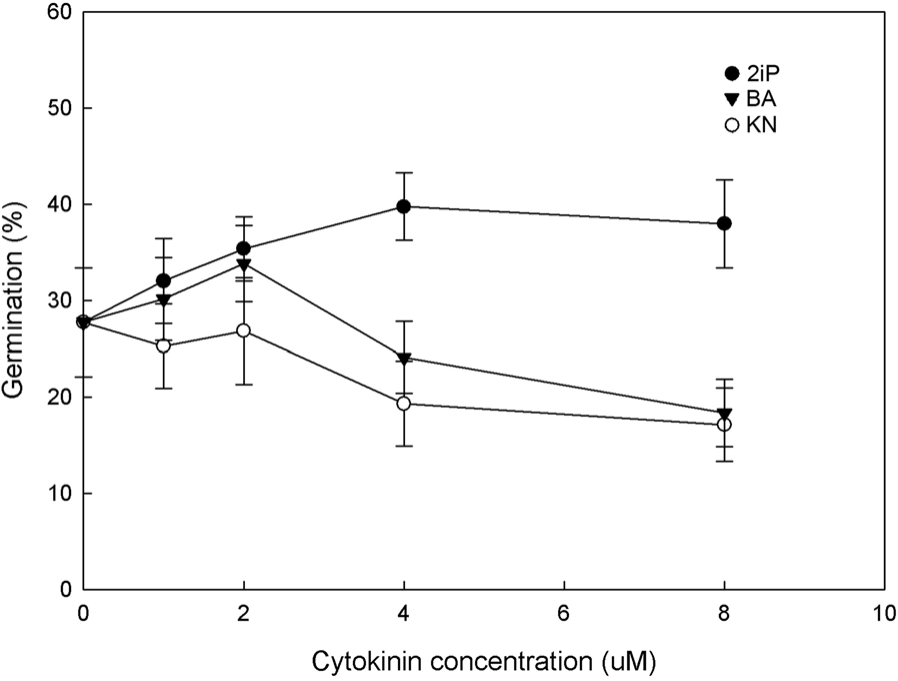
Effects of various cytokinins on seed germination of *C. subtropicum* after 6 months of culture. Error bars represent SE (n = 3)

**Fig. 6:**
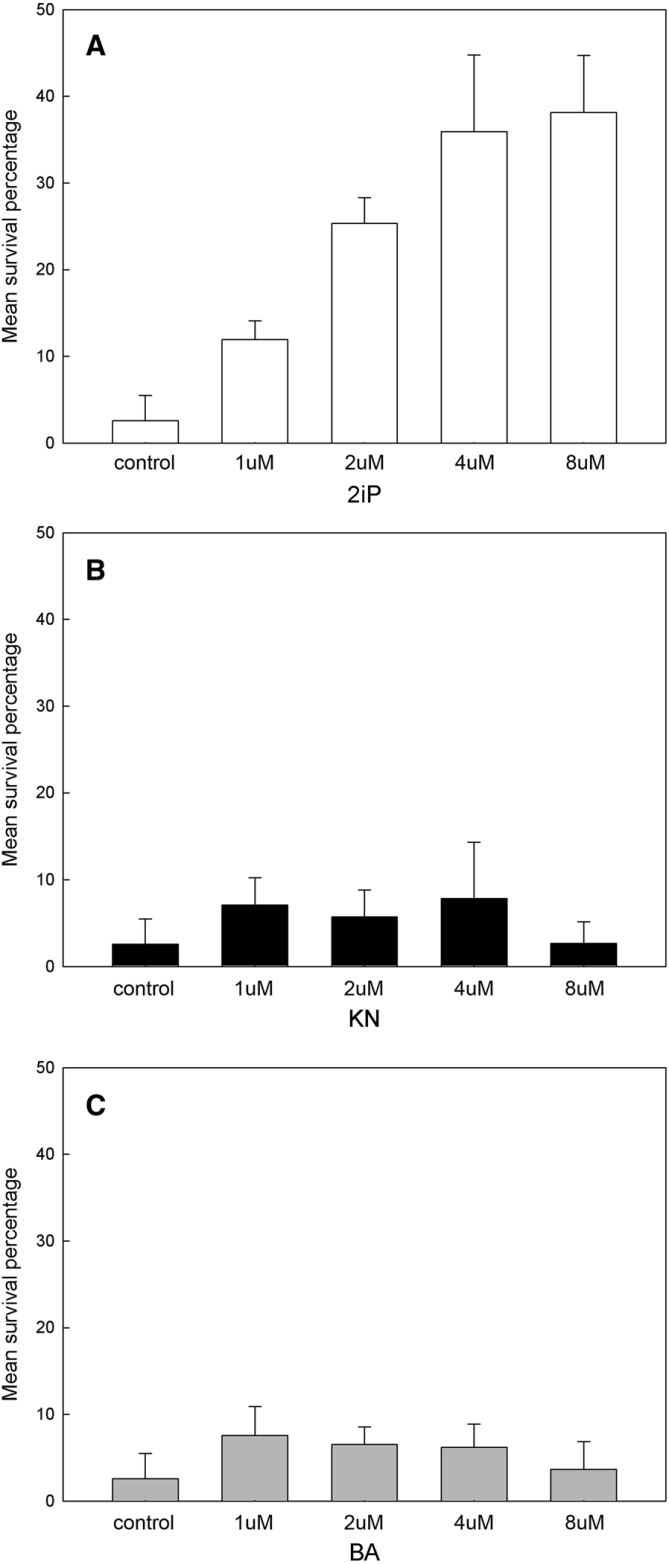
Effects of various cytokinins on the survival percentage of *C. subtropicum* protocorms after 6 months of culture. **A** 6-(γ,γ-Dimethylallylamino)purine (2iP). **B** Kinetin (KN). **C** 6-Benzylaminopurine (BA). Error bars represent SE (n = 3)

The germination percentage was affected by the addition of different types of cytokinins (Fig. 5). Both 2iP and BA stimulated seed germination compared with the control, but higher concentrations of BA (4 and 8 μM) suppressed seed germination. At low concentrations, the addition of KN neither increased nor inhibited seed germination. After germination, several protocorms browned and ultimately died, especially on the culture medium without cytokinins. The survival percentage of protocorms was higher on culture medium containing 2iP than on culture medium containing KN or BA (Fig. 6) and increased with the concentration of 2iP in the culture medium.

### Seedling growth

At 4 months after sowing, the developing young seedlings (Fig. 3D) were transferred to flasks containing modified Norstog medium supplemented with 1 mg l^–1^ malic acid, 20 g l^−1^ sucrose, and 20 g l^−1^ potato homogenate and solidified with 7 g l^−1^ agar. After 3 months of subculture, the seedlings had increased in size, with prominent root formation (Fig. 3E). After 5 months of growth in the greenhouse, the in vitro raised seedlings were transferred to clay pots filled with potting mix. The shoots had sprouted and elongated, and their leaves began to expand (Fig. 3F). Survival of the seedlings with leaf expansion ranged from 90 to 95%. This protocol of in vitro germination and development of healthy seedlings could be helpful for reintroduction of *C. subtropicum* to its natural habitat or propagation as a horticultural product.

## Discussion

We found that germination of *C. subtropicum* was optimal when seeds were collected at 105 DAP (Fig. 4). Our data confirm that the timing of seed collection is critical in optimizing asymbiotic germination (De Pauw and Remphrey 1993; Lee et al. 2005; Zhang et al. 2013; Jiang et al. 2017). The optimal timing of seed collection from *Cypripedium* species may reflect the climate of the natural habitat. *Cypripedium* species in the northern temperate region, such as *C. candidum* (42 DAP), *C. macranthos* (42 DAP), *C. parviflorum* (56 DAP) and *C. reginae* (56 DAP) (see De Pauw and Remphrey 1993 and Zhang et al. 2013), usually develop faster, while species in the subtropical region, such as *C. formosanum* (90–105 DAP) and *C. lentiginosum* (90–105 DAP), tend to have prolonged seed development (see Lee et al. 2005 and Jiang et al. 2017). *C. subtropicum* is distinct from most *Cypripedium* species in that it has nondormant shoots with evergreen leaves and inhabits moist broadleaf forests in subtropical to tropical zones (Jiang and Liu 2009; Averyanov et al. 2017). Taken together, our results and previous reports (Lee et al. 2005; Jiang et al. 2017) suggest that subtropical *Cypripedium* species may be adapted to warmer climates and have prolonged seed development because of the long growing season.

Interestingly, *C. subtropicum* germination increased remarkably beginning with seeds collected at 90 DAP. Germination peaked for seeds collected at 105 DAP (Fig. 4) and decreased sharply for seeds collected after 120 DAP. Histological analysis showed that at 105 DAP, the embryo had ceased cell division and was beginning to mature, with accumulation of storage products within the embryo proper (Fig. 2F). At this stage, suspensor degeneration occurred, and the inner and outer seed coats were moist and not compressed. These findings are consistent with previous structural observations that the germination percentage of orchid seeds is higher when the seed coat has not yet acquired its strong hydrophobic nature and barriers to rapid rehydration are limited (Rasmussen 1995). By 105 DAP, as the seed approached maturity, both the inner and outer seed coats had compressed into thin layers (Fig. 2G). The compressed inner seed coat formed an impermeable layer (also known as a carapace) enveloping the embryo. This layer has been implicated in the control of seed dormancy in several terrestrial orchids (Lee et al. 2005; Yamazaki and Myoshi 2006).

During *C. subtropicum* seed development, the outer seed coat elongated considerably, resulting in a hair-like appearance of the mature seeds (approximately 4 mm long and 0.2 mm wide; Fig. 3A). Most *Cypripedium* species possess a fusiform seed coat (Eccarius 2009), and the hair-like appearance of the seed coat of *C. subtropicum* is unique. The inflorescence stalk of *C. subtropicum* is 1 m tall, and as the capsules ripen and dehisce to release seeds, the long hair of the seed coat may facilitate seed dispersal via wind in the shady understory forest layer. Histochemical analysis showed that both the inner and outer seed coats had a greenish blue color upon TBO staining, suggesting the presence of lignin in their walls (Fig. 2G). Moreover, Nile red staining indicated the presence of cuticular material in the surface wall of the embryo proper (Fig. 2H). The walls of both the inner and outer seed coats fluoresced only weakly, and the fluorescence was quenched by prestaining with TBO, implying the absence of distinct cuticular material in the walls of the inner and outer seed coats (see Holloway, 1982; Yeung et al. 1996). By contrast, we previously detected distinct cuticular material in the walls of the seed coat of *C. formosanum* (Lee et al. 2005). Using ATR-FT-IR spectroscopy, Barsberg et al. (2018) observed differential seed coat accumulation of C-lignin and lipids between *C. calceolus* and *C. formosanum*. Taken together, these results indicate that there is a rich diversity of seed coat chemistry among *Cypripedium* species, leading to varying seed coat permeability.

### Cytokinins and seed germination

Arditti and Ernst (1984) reported that the influences of cytokinins on seed germination vary depending on the orchid species. In *Cypripedium*, the application of cytokinins has been shown to promote germination in vitro (van Waes and Debergh 1986), and preferences for different types of cytokinins or a particular cytokinin for seed germination have been reported for various *Cypripedium* species. KN has the strongest stimulatory effect on *C. regina* germination, followed by BA and 2iP (Harvais 1982). By contrast, BA and 2iP both promote *C. candidum* germination, while KN has little effect (De Pauw et al. 1995). In *C. macranthos*, BA, thidiazuron (TDZ), zeatin, 2iP, and KN all promote germination, while N-(2-chloro-4pyridyl)-N′-phenylurea (4PU) does not; KN is the most effective (Miyoshi and Mii 1998). In *C. lentiginosum*, only 2iP enhances germination, while BA, kinetin and TDZ have suppressive effects (Jiang et al. 2017). In the present study, BA and 2iP both improved *C. subtropicum* germination, but only BA enhanced germination at lower concentrations (less than 4 μM) (Fig. 5). Taken together, these results suggest that the sensitivity of seed germination to different cytokinins is species specific. Notably, germination of *Cypripedium* seeds in the absence of exogenous cytokinins has been observed; the ungerminated seeds were likely deficient in endogenous cytokinins (De Pauw et al. 1995). High levels of endogenous abscisic acid (ABA) have been detected in mature seeds of terrestrial orchids such as *Calanthe* (Lee et al. 2007), *Cypripedium* (Lee et al. 2015), *Epipactis* and *Dactylorhiza* (Van der Kinderen 1987). Exogenous cytokinin supplementation may overcome the inhibitory effect of ABA and thus stimulate seed germination (Black et al. 1974).

For many terrestrial orchids, browning and subsequent death of the protocorm are commonly observed after seed germination (Stoutamire 1974; Harvais 1982). In *C. subtropicum*, 2iP improved the protocorm survival rate, while KN and BA had little effect (Fig. 6). De Pauw et al. (1995) found that exogenous cytokinins do not prevent *C. candidum* protocorm death. Protocorm death may be due to nutrient imbalance or insufficient growth-stimulating materials in the culture medium (Stoutamire 1974). In *C. acaule*, the degenerated protocorm usually lacks a well-differentiated meristematic region (Leroux et al. 1995). During orchid protocorm development, the meristematic region maintains active mitotic cell division, which is responsible for the formation of the shoot apical meristem required for plant development (Yeung 2017; Yeung et al. 2018). Since cytokinins are essential for functional identify of meristem cells and meristem maintenance (Doerner 2007), our results suggest that the presence of cytokinins, especially 2iP, is necessary for the normal growth and development of *C. subtropicum* protocorms.

## Conclusions

In this study, we investigated the major structural features of *C. subtropicum* seed development and the optimal timing of seed collection for germination in vitro. For culturing immature seeds on modified Norstog medium, the optimum time of seed collection was 105 DAP (at the globular embryo stage). Supplementation with 2iP improved seed germination and the protocorm survival rate. This study provides a scientific basis for seedling establishment through asymbiotic seed culture for further reintroduction efforts.

## Data Availability

Not applicable.
